# Effective management of attention-deficit/hyperactivity disorder (ADHD) through structured re-assessment: the Dundee ADHD Clinical Care Pathway

**DOI:** 10.1186/s13034-015-0083-2

**Published:** 2015-11-19

**Authors:** David Coghill, Sarah Seth

**Affiliations:** Division of Neuroscience, Ninewells Hospital and Medical School, University of Dundee, Dundee, DD1 9SY UK

**Keywords:** Attention-deficit/hyperactivity disorder, Titration, Treatment response, Inadequate response

## Abstract

Attention-deficit/hyperactivity disorder (ADHD) has become a major aspect of the work of child and adolescent psychiatrists and paediatricians in the UK. In Scotland, Child and Adolescent Mental Health Services were required to address an increase in referral rates and changes in evidence-based medicine and guidelines without additional funding. In response to this, clinicians in Dundee have, over the past 15 years, pioneered the use of integrated psychiatric, paediatric, nursing, occupational therapy, dietetic and psychological care with the development of a clearly structured, evidence-based assessment and treatment pathway to provide effective therapy for children and adolescents with ADHD. The Dundee ADHD Clinical Care Pathway (DACCP) uses standard protocols for assessment, titration and routine monitoring of clinical care and treatment outcomes, with much of the clinical work being nurse led. The DACCP has received international attention and has been used as a template for service development in many countries. This review describes the four key stages of the clinical care pathway (referral and pre-assessment; assessment, diagnosis and treatment planning; initiating treatment; and continuing care) and discusses translation of the DACCP into other healthcare systems. Tools for healthcare professionals to use or adapt according to their own clinical settings are also provided.

**Electronic supplementary material:**

The online version of this article (doi:10.1186/s13034-015-0083-2) contains supplementary material, which is available to authorized users.

## Background

Attention-deficit/hyperactivity disorder (ADHD) is a heterogeneous neurodevelopmental disorder with a worldwide prevalence of 5–7 % in children and adolescents [1, 2]; UK prevalence is estimated at 2.2 % [3]. The disorder is characterized by core symptoms of inattention, hyperactivity and impulsivity [4, 5], and is associated with functional impairment [6–8]. In the UK, ADHD management is primarily the responsibility of specialists based within either paediatric departments or Child and Adolescent Mental Health Services (CAMHS). As a consequence of an increase in awareness and acceptance of ADHD in the UK in recent years, management of this disorder has become a major aspect of the work of these services [9, 10]. This has required adaptations, usually within existing budgets and staffing levels, to accommodate this increased workload.

In a 5-year study, most adolescents with ADHD managed in a UK community setting had continuing difficulties despite contact with CAMHS and pharmacotherapy [11]; the authors of this report concluded that “the treatment and monitoring of ADHD need to be intensified” [11]. This concurs with the findings of the Multimodal Treatment Study of Children with ADHD (MTA) [12, 13], which showed that a carefully implemented approach to medication is superior to routine clinical care. However, the use of symptom thresholds or specific impairment criteria during ADHD assessment, or standardized or systematic criteria to assess treatment outcomes is still limited within UK clinical settings [14, 15].

ADHD treatment guidelines and algorithms, including those for England and Wales [16], Scotland [17, 18], Europe [19–25], and North America [26–28], have proposed evidence-based approaches for ADHD management. However, tools to translate this guidance into everyday clinical practice are lacking. While Hill and Taylor published an auditable protocol for treating ADHD in 2001 [29] and CADDRA published several toolkits to support ADHD practitioners, we are unaware of any other detailed descriptions of effective, evidence-based pathways that have been developed and implemented within a real-world setting. Therefore, we developed an implementable evidence-based clinical pathway for the assessment and management of ADHD. Here, we describe the pathway and provide the protocols and supporting tools necessary for wider use. We hope that the information provided will be adapted by others to suit their local healthcare service structure and resources.

## The Dundee ADHD Clinical Care Pathway

Dundee and Angus are Scottish regions with a broad sociodemographic composition, including urban and rural areas of both considerable social deprivation and relative affluence. Specific clinical services for ADHD in the region are managed by the National Health Service (NHS) generic CAMHS service and delivered by non-academic NHS clinicians. Over the last 15 years, Dundee CAMHS has developed a clearly structured, evidence-based clinical pathway for the assessment and management of children and adolescents with ADHD in Dundee and Angus based on key clinical practice guidelines and other publications (Table 1).

**Table 1:** Key clinical practice guidelines and other publications used in the development of the DACCP

Guidelines
The Scottish Intercollegiate Guidelines Network [17, 18]
National Institute for Clinical Excellence guidelines [16, 30, 31]
Quality Improvement Scotland/Healthcare Improvement Scotland [15, 54, 61]
European guidelines [19–25, 62]
Guidelines and resources from the Canadian Attention Deficit Hyperactivity Disorder Resource Alliance [59]
The Multimodal Treatment Study of Children with ADHD [12, 13, 63–66]
Texas Children’s Medication Algorithm [67, 68]
Scottish Medicines Consortium and National Institute for Clinical Excellence advice on the use of lisdexamfetamine [69, 70]

*ADHD* attention-deficit/hyperactivity disorder, *DACCP* Dundee ADHD Clinical Care Pathway

The Dundee ADHD Clinical Care Pathway (DACCP) was developed to facilitate the dynamic integration of new knowledge in order to provide effective, evidence-based therapy; speed up the transfer of research findings into clinical practice; use staff skills and time effectively; and provide a consistent approach to the management of waiting lists and treatment. The DACCP integrates psychiatric, paediatric, nursing, occupational therapy, dietetic and psychological care. A key focus of the pathway is the routine use of standardized protocols for the assessment, titration and monitoring of clinical care. These protocols incorporate accessible, free or low-cost, clinically relevant, well-validated instruments at all stages of the pathway. The use of clinical outcome assessments to inform day-to-day clinical decision-making is particularly important, and is in keeping with key findings from the MTA study [12, 13].

The pathway is dynamic and in continuous development; up-to-date, evidence-based approaches to assessment and treatment are implemented into the DACCP as quickly as possible. While clinical care is delivered within a non-academic, clinical setting, there are close ties with the University of Dundee, where staff are heavily involved in the generation and evaluation of new evidence to advance the management of ADHD and in the development of clinical guidelines. These associations have undoubtedly played an important part in the development and implementation of the pathway. However, we believe that having now developed and refined the pathway over several years it is now ready to be implemented in broader settings.

Approximately 800 patients (~1.2 % of the local school-age population) currently receive care via the DACCP. The pathway was formally evaluated in the 2012 Scotland-wide audit of ADHD by Health Improvement Scotland [15]. This audit found the DACCP to be compliant with all of the major recommendations of the Scottish Intercollegiate Guidelines Network (SIGN) [18] and the National Institute for Clinical Excellence (NICE) [16, 30, 31] for the assessment and management of ADHD. The pathway was highly praised because it demonstrated the provision of robust, quality-based, protocol-driven and non-profession-specific clinical care [15]. It was also the only ADHD pathway in Scotland that routinely measured clinical outcomes [15]. The pathway has received international attention and has been used as a template for service development in many countries (personal communication, D Coghill).

## Stages of the DACCP

**Fig. 1:**
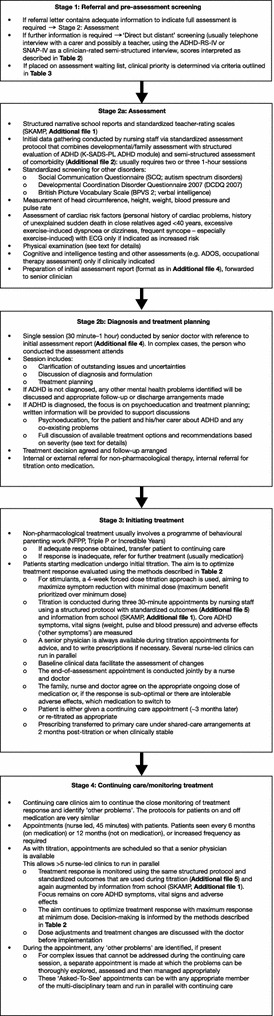
Flow diagram showing the four stages of the Dundee ADHD Clinical Care Pathway. *ADHD* attention-deficit/hyperactivity disorder, *ADHD-RS-IV* attention-deficit/hyperactivity disorder rating scale IV, *ADOS* Austistic Diagnostic Observation Schedule, *ECG* electrocardiogram, *K-SADS-PL* Schedule for Affective Disorders and Schizophrenia for School-Age Children-Present and Lifetime version, *NFPP* New Forest Parenting Programme, *SKAMP* Swanson, Kotkin, Agler, M-Flynn and Pelham scale, *SNAP-IV* Swanson, Nolan and Pelham-IV questionnaire

The pathway has four key stages, described in detail below, and summarized in Fig. 1.

### 1. Referral and pre-assessment screening

In approximately 80 % of cases, the information in the referral letter is adequate to decide whether a full clinical assessment is warranted. Where insufficient information is provided (e.g. clinical problems are unclear or do not indicate whether impairment is likely), a ‘direct but distant’ approach is used to obtain additional insight whenever possible, as it combines accuracy with efficient resource use. Telephone interviews are conducted with a parent/carer, followed by a teacher if necessary. These are typically conducted by a specialist nurse using either the ADHD rating scale IV (ADHD-RS-IV) or the ADHD questions from the Swanson, Nolan and Pelham (SNAP)-IV questionnaire, delivered as a clinician-rated semi-structured interview (Table 2). A mean item score (total or sub-scale) of >2 is highly suggestive of ADHD; intermediate scores (1–2) require clinical judgement. This approach combines good sensitivity (83 %) and better specificity (97 %; i.e. fewer false positives) compared with the indirect questionnaire-based approach outlined below (unpublished observations, D Coghill).

**Table 2:** Clinical interpretation of scores from the ADHD-RS-IV or the SNAP-IV questionnaires

ADHD-RS-IV or SNAP-IV questionnaire score				(i) Pre-assessment screening		(ii) Post-treatment monitoring
Total score (range 0–54)	Mean item total score^a^	Subscale^b^ score (range 0–27)	Mean item subscale score^a^	Clinical interpretation	Clinical outcome	Clinical interpretation
0–18	≤1	0–9	≤1	ADHD unlikely. Alternative explanations for clinical presentation to be considered	Refer to other CAMHS divisions or paediatrics for assessment of non-ADHD problems OR discharge and/or refer to another agency (e.g. social work or education)	Very good/optimal response: symptoms well within normal range
19–26	<1.5	10–13	<1.5	May require full assessment. Decision based on clinical judgement using all available evidence	Outcome depends on qualitative assessment and clinical judgement. Full assessment is scheduled to exclude or confirm ADHD, as below. If ADHD is considered unlikely, refer to other CAMHS divisions OR discharge and/or refer to another agency, as above	Good response: symptoms within normal range but may be improved
27–36	1.5–2	14–18	1.5–2	May require full assessment. Use clinical judgement based on all available evidence		Response still clinically significant: symptoms within normal range but response probably inadequate. Need to assess other factors
37–54	>2	19–27	>2	ADHD likely. Needs full assessment	Conduct a full assessment (see text for details: DACCP Stage 2)	Inadequate response: many symptoms still observed. Need to assess other factors

Clinical interpretation of scores from the ADHD-RS-IV, or ADHD questions from the SNAP-IV questionnaire, when used as (i) a pre-assessment screening tool or (ii) post-treatment to monitor treatment response

*ADHD* attention-deficit/hyperactivity disorder, *ADHD-RS-IV* attention-deficit/hyperactivity disorder rating scale IV, *CAMHS* Child and Adolescent Mental Health Services, *DACCP* Dundee ADHD Clinical Care Pathway, *SNAP-IV* Swanson, Nolan and Pelham-IV

^a^Calculated by dividing the total/subscale score by the number of items (18 for the total; 9 for each subscale)

^b^Inattention or Hyperactivity/Impulsivity subscales

Within the DACCP, we focus on this ‘direct but distant’ approach; however, where this is not feasible there are alternative approaches available for pre-screening of referrals, including: indirect contact (e.g. parent-completed questionnaires, such as the generic Strengths and Difficulties Questionnaire [32], or the ADHD-specific Conners [33], ADHD-RS-IV [34] or SNAP-IV [35] questionnaires); and personal assessment using a triage approach or the Choice appointments associated with the Choice and Partnership Approach (CAPA) model [36].

Once a decision has been made to conduct a full assessment, we do not usually request any further pre-assessment parent- or self-completed ADHD questionnaires.

Of note, population-based screening in the DACCP is not utilized. In areas where ADHD is under-diagnosed, such as Scotland [15], the main purpose of screening is to ensure that patients do not go unrecognized. However, population-based approaches using parent- and/or teacher-rated questionnaires are associated with high false positive rates [37].

#### Waiting list prioritization

Complex neurodevelopmental disorders (such as ADHD, autism spectrum disorders, tic disorders and Tourette’s syndrome, as well as learning disorders and intellectual impairment) can have a dramatic impact on home and family life and it is not uncommon to receive requests for prioritization of care. These cases, however, typically require different criteria for prioritization to other psychiatric disorders. Without appropriate prioritization, those with developmental disorders are at risk of remaining at the end of the queue. Our service therefore runs two parallel prioritization systems (one for ‘emotional disorders’ and one for ‘developmental disorders’), each with its own prioritization criteria. Examples of prioritization criteria for patients with a developmental disorder are shown in Table 3. Within the DACCP, decisions about prioritization are typically conducted by specialist nurses, with backup from senior medical staff as required.

**Table 3:** Priority waiting list: factors indicating the prioritization of a patient with a development disorder

Trigger for prioritization
Placement (with own family or out-of-family) at significant risk of breakdown *and* seeing the patient may reduce this risk *and* social workers are already appropriately involved
Significant health risk will ensue for a patient’s caregiver and/or family members if the patient does not receive treatment
Patient at risk of significant, deliberate self-harm
Patient at significant risk of developing an impairing comorbid disorder (not oppositional defiant disorder or conduct disorder)
Substantial reduction in school attendance has occurred due to multiple or extended exclusions *or* the patient has significantly reduced access to educational opportunities: e.g. a long-term part-time timetable *or* patient can only be taught 1:1 *and*, in all cases, appropriate educational measures are already in place
Patient approaching upper age-limit of the service (≥15.5 years for Dundee CAMHS)

These criteria were designed to identify the ~10 % of patients with the most immediate needs. Patients from priority and routine waiting lists are routed into the assessment process in a 1:1 ratio; however, this ratio could be altered in favour of either waiting list depending on demand

*CAMHS* Child and Adolescent Mental Health Service

### 2. Assessment, diagnosis and treatment planning

The DACCP has developed a standardized protocol for assessment, diagnosis and treatment planning, whereby initial information gathering is conducted by specialist nursing staff, restricting the role of the doctor to diagnosis and treatment planning. This facilitates effective use of limited clinical resources, improving clinical flow.

#### 2a. Information gathering

The focus at this stage is to collect the information required to make a diagnosis and to plan treatment. Clinical information is primarily gathered from parents/carers using a standardized procedure that, in addition to ADHD, also considers potential differential diagnoses and comorbid mental and physical health problems. An interview with the child, focusing on impairment and functioning, is also conducted. Structured narrative school reports and teacher-rating scales, most frequently the Swanson, Kotkin, Agler, M-Flynn and Pelham (SKAMP) scale [38] (Additional file 1), are requested prior to the first assessment visit.

Initial information gathering is completed during one or more face-to-face clinical assessment visits using a structured assessment document (Additional file 2). Presenting problems, health and developmental history, and global functioning are documented, in addition to comorbid psychiatric conditions and any issues in the patient’s family life, social functioning (including peer relationships, criminal behaviour, etc.) and school functioning. Within the DACCP, this assessment is conducted by a core CAMHS worker (a nurse, primary mental health worker^1^ or clinical psychologist); all staff are trained in all aspects of the assessment.

A structured assessment of ADHD is performed using the ADHD section of the Schedule for Affective Disorders and Schizophrenia for School-Age Children-Present and Lifetime version (K-SADS-PL) [39, 40]. Additional routine screening questions cover the full range of mental health problems, including; autism spectrum disorders, developmental communication disorders and social communication disorder. Standardized screening questionnaires (summarized in Additional file 3) are used to support the identification of common co-existing disorders.

A general physical examination, including observation of the standard of general care, assessment for stigmata of congenital disorders and neurodevelopmental immaturity, a vision and hearing check, a screen of gross and fine motor functioning and a screen for motor and vocal tics, is suggested during the initial assessment. Physical health (head circumference, height, weight, blood pressure and pulse rate) and assessment of cardiac risk factors are recorded at assessment (and routinely thereafter). In line with guideline recommendations, routine blood tests, electroencephalography or electrocardiography are not routinely conducted, unless there is a specific indication [20, 23, 24].

Following the interview, additional information (e.g. from the patient’s school or other agencies) is requested as required. Patients may be referred for additional specific assessments (e.g. the Autistic Diagnostic Observation Schedule for autism [41], occupational therapy for developmental coordination disorder and/or sensory sensitivity, cognitive testing or paediatric assessment for physical problems). While cognitive and neuropsychological testing are not part of the routine assessment, the British Picture Vocabulary Scale [42] is utilised routinely as an estimate of verbal intelligence.

#### 2b. Diagnosis and treatment planning

Once the required information has been gathered, a standardized assessment report (Additional file 4) is compiled and forwarded to a senior clinician (usually a consultant or associate specialist/higher specialist trainee), who will review the information and arrange an “end of assessment” appointment with the patient and their family to discuss diagnosis and treatment planning. Whilst it is often possible to conclude the ADHD assessment while awaiting the outcome of additional information, it is sometimes necessary to delay this meeting until all data are available. The core CAMHS worker who conducted the initial assessment does not usually need to attend, but this may be helpful in complex cases. At this meeting, the consultant does not spend valuable time revisiting issues that have been adequately covered during the assessment; rather he/she aims to address any outstanding uncertainties, provide a diagnosis and formulation and agree a management/treatment plan.

Both the International Classification of Diseases (ICD-10) [5] and the Diagnostic and Statistical Manual of Mental Disorders 5 (DSM-5) [4] definitions of hyperkinetic disorder (HKD)/ADHD are considered during diagnosis, respectively. The ICD definition of HKD is more restrictive than DSM-defined ADHD and requires that inattentive, hyperactive and impulsive symptoms are all present and are both pervasive and impairing. While symptoms must also be pervasive and impairing in DSM-defined ADHD, the requirements are less strict and DSM-defined ADHD includes less severe cases than HKD [4, 5]. If ADHD or HKD is diagnosed, the focus for the remainder of the meeting is to provide psychoeducation about ADHD and any co-existing problems, and to discuss the various treatment options available. Written information and suggestions for web-based support materials are provided to support these discussions.

Initial treatment decisions generally follow the recommendations of the SIGN [18], NICE [16, 30, 31] and European guidelines [20, 21, 23–25]. Initial therapy depends on symptom severity, circumstances, comorbidities, patient preference and parent/carer preference [16], and usually includes recommendations for school interventions. Treatment options include non-pharmacological interventions and pharmacotherapies.

If ADHD is not diagnosed, any other mental health problems that have been identified will be discussed and appropriate arrangements made for follow-up or discharge.

### 3. Initiating treatment

**Fig. 2:**
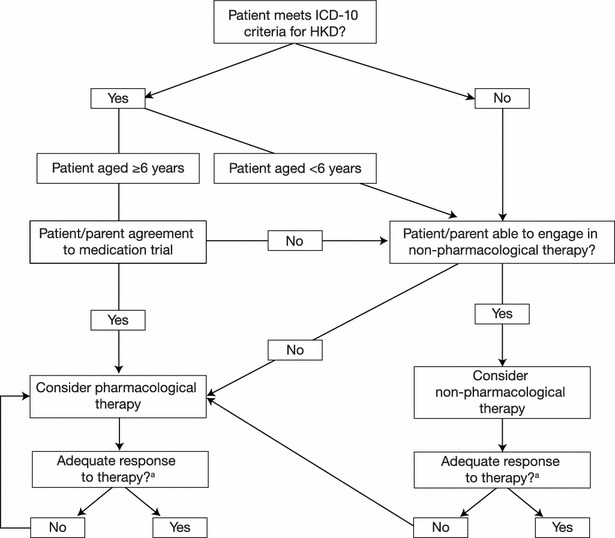
DACCP treatment algorithm: selection of pharmacological versus non-pharmacological therapy for patients with ADHD. ^a^For the evaluation of treatment response, please refer to section ‘How do we define optimal/adequate/inadequate response?’. For non-pharmacological therapy, treatment response is reviewed at the end of a course of therapy (programmes are usually 10–12 sessions) and annually thereafter. The use of medication as first-line treatment does not preclude combining this with a non-pharmacological approach]. *ADHD* attention-deficit/hyperactivity disorder, *DACCP* Dundee ADHD Clinical Care Pathway, *HKD* hyperkinetic disorder, *ICD* International Classification of Diseases

The initial focus of treatment is to reduce the core symptoms of ADHD. Medication is usually offered as first-line treatment for patients aged 6 years and over who meet ICD-10 criteria for HKD (Fig. 2). Non-pharmacological treatment, consisting primarily of parenting interventions that focus on behavioural management, is generally recommended for children under 6 years of age, those who meet DSM criteria for ADHD but not ICD criteria for HKD and those whose parents are resistant to medication options. Parenting programs readily available in Dundee include: New Forest Parenting Programme (NFPP) [43], Triple P [44] and Incredible Years [45]. If the treatment response to a parenting intervention is considered adequate, the need for additional interventions to address any remaining difficulties is assessed and follow-up in the continuing care clinic is arranged (see below for further details). If the treatment response is inadequate, further treatment options are discussed; typically involving medication.

#### Initiating and titrating medication for ADHD

##### Initial medication options

The choice of first-line medication is informed by clinical guidelines [16–18, 20, 21]. In most cases, a stimulant medication is the first choice and methylphenidate is most commonly prescribed. Primary school-age children (up to 11 years) usually begin treatment with immediate-release methylphenidate (which is less expensive—a priority for publically funded services—more flexible and has a short duration of adverse events), whereas older children usually start with a long-acting formulation (which is less stigmatizing and has a lower risk of diversion as medication is not taken during school hours). For patients with tic disorders, issues with substance misuse or a strong family preference to avoid stimulants [16], atomoxetine may be considered as a first-line treatment.

##### Dose titration

As informed by the MTA study and in line with clinical guidelines, the DACCP places considerable importance on accurate dose titration, with the aim of achieving maximum benefit with minimal adverse effects. Maximum benefit is prioritized over minimum dose. A 4-week, structured dose-optimization schedule is used for all patients prescribed immediate-release stimulants or extended-release methylphenidate. The dose is increased from 5 to 20 mg three times per day for immediate-release formulations or equivalent dose for long-acting formulations. Medication is usually initiated with 12-h cover, 7 days a week, without routine drug holidays.

Baseline and titration appointments are nurse led (although a senior clinician is always available for advice and to write prescriptions, if required) and last approximately 30 min. During the baseline appointment, patients are informed of the purpose of titration, the schedule is agreed and baseline assessments performed (see below). Three or four titration appointments are typically required, depending on the medication and clinical response. Titration appointments are conducted face-to-face or by telephone (in which case local health services may need to perform weight, pulse and blood pressure assessments). The patient is reviewed jointly by a nurse and a physician at the end of the 4-week period and they, in discussion with the family, agree on the ongoing medication and dose.

In addition to clinical feedback from the patient and parent/carer, the following information is gathered using standardized documentation at baseline and each subsequent titration appointment (Additional files 1, 5):

- ADHD-RS-IV or SNAP-IV, administered as a semi-structured interview and rated by the clinician.
- SKAMP report, completed by the patient’s teacher.
- Clinical Global Impression-Severity and -Improvement rating scales.
- Children’s Global Assessment Scale.
- Structured assessment of ‘other symptoms’. Although the purpose is to identify treatment-related adverse effects, we ask patients ‘Do you have these symptoms?’ and ‘Are they impairing?’ rather than ‘Did medication cause these problems?’. The clinician then decides whether any identified symptoms are likely related to medication or the underlying ADHD.
- Weight, blood pressure and pulse rate.

##### Assessment of symptom control and tolerability

Medication doses are increased at each visit, unless symptoms are already under optimal control (indicated by a mean post-treatment score of ≤1 for ADHD-RS-IV or the ADHD questions from SNAP-IV; see section on defining adequate/inadequate response below and Table 2) or there are significant adverse effects. When symptom control is considered optimal, the end-of-titration appointment is usually brought forward and the dose maintained. The patients exiting titration are booked into a continuing care clinic approximately 3 months later, and prescribing, but not monitoring, is transferred relatively quickly to primary care under a shared-care agreement.

If a patient experiences adverse effects, the dose is usually decreased, but may be either continued for another week or increased as originally scheduled to assess treatment benefit versus adverse effects.

If there has been no clinical response to a maximum dose (usually 20 mg methylphenidate tds or equivalent) or the patient has experienced significant adverse effects, switching to an alternative medication or a different approach is considered (described further below). A full discussion of the management of adverse effects is beyond the scope of this article; interested readers are directed to Cortese et al. [23] for further information.

#### How do we define optimal/adequate/inadequate response?

Individual response to ADHD therapy is influenced by a number of factors, including severity of the disorder, sensitivity to a specific treatment, vulnerability to treatment-related adverse effects, and personal values and preferences regarding treatment outcome [46]. Indeed, the perception of treatment response is subjective and thus may differ depending on the reporter. In the DACCP, information on treatment response is always gathered from both the patient and the parent/carer, using a semi-structured interview. During titration, good symptom control is considered the key outcome by the DACCP.

Using a combination of clinical data, published norms, the results of clinical trials and established statistical methods [47], we calculated a clinically meaningful cut-off score for the ADHD-RS-IV when used as a semi-structured interview. This, combined with clinical experience and published data, has suggested scores associated with different clinical states. The mean (standard deviation [SD]) ADHD-RS-IV total score for untreated individuals with ADHD was reported as 41.8 (8.3) in the UK [48]. In general, a decrease in total score of >11 from baseline suggests a clinically meaningful response. As the ADHD-RS-IV and the ADHD section of SNAP-IV are very similar, it seems likely that the same scoring rules can be applied to SNAP-IV.

The clinical significance of post-treatment reductions in ADHD-RS-IV and SNAP-IV scores are thoroughly described in Table 2. Although these definitions are used to guide clinical decision-making, they must be applied flexibly, and the final judgement of the adequacy of treatment response requires clinical judgement and consideration of all available information.

#### Treatment switching

Of those children with ADHD, 70–80 % respond well to either methylphenidate or d-amphetamines and 90–95 % respond to at least one class of stimulant [49–53]. Where a patient is judged to have an inadequate clinical response to methylphenidate at the end of titration, switching to lisdexamfetamine or atomoxetine is usually recommended and the titration process repeated. Titration of lisdexamfetamine is similar to that of methylphenidate, but with three rather than four dose steps (30, 50 and 70 mg). Titration of atomoxetine begins with a dose of 0.5 mg/kg for 1 week, then increased to 1.2 mg/kg for at least 12 weeks (unless there are intolerable adverse effects) to fully assess the benefits. The dose is increased to 1.8 mg/kg if there is only a partial response.

### 4. Continuing care/monitoring treatment

Although titration and optimization of the initial response to medication are important, data from the MTA suggest that close attention to continuing care is also essential [12]. Accordingly, all patients on the DACCP, regardless of medication status, are followed up. The purpose of continuing care clinics is to monitor and adjust ADHD treatments and to identify any ‘other problems’ that will require additional sessions for further assessment or treatment [12]. Continuing care clinics are nurse led but a senior clinician (consultant or associate specialist/higher specialist trainee) is always available to discuss proposed changes to treatment, review patients with particularly complex issues and/or discuss stable patients who do not require changes to care after the clinic has finished. Clinics are conducted by the patient’s core worker if possible for continuity of care. Each appointment is scheduled for 45 min. Up to six clinics are held simultaneously to make the best use of senior clinicians’ time.

For patients receiving medication, the typical interval between review appointments is 6 months; however, more frequent appointments are available as necessary. Annual reviews are conducted for patients receiving non-pharmacological interventions. Patients who are not being actively treated are also followed up at least annually as it is not uncommon for these patients to experience renewed difficulties, especially at times of transition (e.g. moving from primary to secondary school) or stress (e.g. periods of family discord).

Continuing care clinics use the same structured data collection instruments and standardized assessment tools used during medication titration (Additional file 5). However, there is a change of emphasis to collect information on medication issues (such as breakthrough symptoms), adherence and stigmatization, in addition to the standard clinical outcomes collected during titration. During this treatment phase, we also placed increased emphasis on the broader picture, such as comorbid mental health issues, physical problems, learning difficulties, ongoing functional impairment and quality of life, including peer and family relationships, school and academic progress and social life. Identified issues are assessed using standardized instruments and assessments as appropriate (Additional file 3).

The identification of these ‘other problems’ is the key to providing good quality holistic care for patients with ADHD. Typical issues include:

- assessment of sleeping or eating difficulties
- assessment of mood or anxiety problems
- liaison with schools or other agencies
- assessment of the need for parent training or other psychological interventions
- discussion of complex medication issues
- cognitive testing
- occupational therapy assessment.

Some of the simple problems, such as sleep and eating difficulties, can be managed within the continuing care clinic appointment. However, time constraints mean additional appointments are often required to focus on identified issues. These appointments are arranged either with the core worker or as a specific ‘asked-to-see’ assessment with an appropriate team member (e.g. a clinical psychologist, dietician or physician).

## Outcomes of the DACCP

Clinical pathways need to demonstrate positive outcomes. As noted previously, the DACCP received favourable reviews from the Healthcare Improvement Scotland 2008 and 2012 audits of ADHD services across Scotland [15, 54]. These reflect the DACCP’s implementation of and adherence to the SIGN clinical practice guidelines [18]. In addition, clinical outcomes are routinely reviewed by the DACCP team. For example, from a random sample of 150 patients currently in continuing care, 96 % (144/150) are receiving pharmacological treatment, most commonly methylphenidate (83 %; 119/144), followed by lisdexamfetamine (9 %; 13/144) and atomoxetine (8 %; 12/144). The remaining 4 % (6/150) of patients are unmedicated. Overall, our clinical outcome data support the use of the DACCP and provide evidence that we can replicate improvements in ADHD symptoms observed in clinical trials within a real-world setting. For example, among the 119 patients currently in continuing care and receiving methylphenidate (Table 4), their mean (SD) total ADHD-RS-IV item score at baseline was 2.5 (0.4), and none had a mean item score of ≤1, indicating a severely impaired population (see Table 2 for clinical interpretation of scores). Mean (SD) item score decreased to 0.7 (0.4) at the end of titration (best dose), indicating a strong clinical response and 80 % of patients had a mean item score of ≤1. At the most recent clinic visit, mean (SD) total ADHD-RS-IV item score remained low at 0.8 (0.8), although the average score across all post-titration continuing care visits was slightly higher (1.0 [0.6]). The mean total ADHD-RS-IV score decreased by 29.4 points from baseline to their most recent visit. This is in line with changes in total ADHD-RS-IV scores observed in a rigorously conducted randomized clinical trial of European children and adolescents treated with stimulant ADHD medication for 7 weeks [55]. In this study, the mean (SD) total ADHD-RS-IV scores at baseline for patients treated with lisdexamfetamine or methylphenidate were 41.0 (7.3) and 40.4 (6.8), respectively, and least squares mean reductions (standard error) from baseline to endpoint were 24.3 (1.2) and 18.7 (1.1), respectively [55].

**Table 4:** Clinical outcome data for patients with ADHD in continuing care receiving methylphenidate (random sample; N = 119)

Visit	Time in treatment (months)	MPH dose (mg)	ADHD-RS-IV score, mean (SD)						ADHD-RS-IV total score ≤18 (mean item score ≤1)
			Inattention subscale		Hyperactivity/Impulsivity subscale		Total		
	Mean (SD); range	Mean (SD)	Subscale score	Mean item score^a^	Subscale score	Mean item score^a^	Total score	Mean item score^a^	n (%)
Baseline^b^	n/a	n/a	21.8 (4.3)	2.4 (0.5)	22.4 (4.3)	2.5 (0.5)	44.2 (6.9)	2.5 (0.4)	0 (0)
End of titration (best dose)	n/a	45.3 (14.0)	6.2 (4.1)	0.7 (0.5)	6.2 (4.1)	0.7 (0.5)	12.2 (7.7)	0.7 (0.4)	95 (80)
Most recent clinic visit	n/a^d^	57.0 (19.7)	7.5 (5.9)	0.8 (0.8)	7.1 (6.3)	0.8 (0.8)	14.8 (12.1)	0.8 (0.8)	63 (53)
Continuing care (mean)^c^	43.5 (28.5); 1–119	51.8 (14.4)	9.2 (4.2)	1.0 (0.5)	8.8 (4.6)	1.0 (0.6)	18.0 (8.4)	1.0 (0.6)	57 (48)

Data presented at the 5° Simpósio Perturbação de Hiperatividade e Défice de Atenção, Coimbra, Portugal, 16–17 April 2015, and available online at http://discovery.dundee.ac.uk/portal/files/6693836/optimizing_treatment_for_ADHD_dc.pdf. Included by permission of the author

*ADHD* attention-deficit/hyperactivity disorder, *ADHD-RS-IV* attention-deficit/hyperactivity disorder rating scale IV, *MPH* methylphenidate, *n/a* not available, *SD* standard deviation

^a^Calculated by dividing the total/subscale score by the number of items (9 for each subscale; 18 for the total)

^b^Pre-treatment (all patients were naïve to ADHD medication)

^c^Mean scores over all (post-titration) continuing care visits

^d^Pearson correlation between time in treatment (months) and ADHD-RS-IV subscale and total scores at most recent clinic visit: Inattention, rho = –0.197, p = 0.07; Hyperactivity/Impulsivity: rho = –0.067, p = 0.5; Total score, rho = –0.145, p = 0.1

Furthermore, we found no significant associations between ADHD-RS-IV subscale and total scores with duration of treatment, which ranged from 1 to 119 months, suggesting that with careful management, methylphenidate may be effective for long-term treatment of ADHD symptoms.

## Staff and training

The DACCP is funded by the NHS from the core CAMHS budget and staffed by employees from within the general CAMHS service. Limited resources in the Dundee CAMHS require us to make best use of available staff. Therefore, much of the clinical work is nurse led, which allows multiple clinics to be held simultaneously and streamlines demand on senior clinician’s time.

At present, there are no dedicated ADHD staff members. Each full-time nurse in the service is involved with assessments and dose titrations and provides ongoing continuing care for about 50–70 patients. This accounts for approximately 60 % of their working week. Most nurses leading the DACCP clinics are not qualified to prescribe ADHD medications. Senior medical cover is provided by doctors with specialist training and experience in either child psychiatry or paediatrics, each contributing 1–1.5 days per week, comprising approximately one full-time equivalent. All clinicians working within the DACCP have had prior experience in general child and adolescent mental health or paediatrics. Junior doctors (doctors in training) are involved when available, and contributions from clinical psychology, occupational therapy and a dietician are made as required.

A multidisciplinary team of experienced clinicians provide supervision and training to new and junior staff on the assessment and management of ADHD, recognition and assessment of common coexisting difficulties, and measurement of clinical outcomes. All new staff members receive formal classroom training on how to conduct assessments, dose titration and continuing care appointments, and the use of standardized instruments to evaluate clinical outcomes. However, most training is conducted within the clinic by observation of consultations with senior nursing medical staff; new staff shadow an experienced clinician until considered competent to work independently. The training period lasts up to 3 months for nurses and typically around 4 weeks for junior doctors. All staff are updated when new information on ADHD becomes available.

## Translation of DACCP into other healthcare systems

The DACCP has proved to be robust in the face of substantial changes to the CAMHS service. Each successive organizational framework has presented challenges. For example, the workflow-based CAPA model [36] was not designed to incorporate the volume of patients seen by ADHD services and, in direct contrast to our pathway, tends to emphasize quantity over quality. We are currently reviewing the implementation of CAPA and it is likely that ADHD care will move out of the CAPA workflow and run in parallel; a move that would be strongly supported by the authors.

The pathway has continued to develop in light of new evidence, experience and ideas from staff. The ethos within the pathway is to be change-orientated and problem-solving in its approach. Changes are often implemented as a result of new findings in the literature or the licensing of a new treatment, but frequently also suggested by a team member and then problem solved by the team, implemented, reviewed and audited, with further changes made as required. Examples of changes include; the adoption of a slimmed down approach to titration appointments to ensure that time is used efficiently during this stage of treatment; the development of an electronic version of the clinic documentation that interfaces with the electronic patient record and facilitates comparison of treatment outcomes and vital signs over time; the implementation of titration protocols for new medications (e.g. the non-stimulants and lisdexamfetamine) that were not available when the pathway was originally designed; and the introduction of locally developed blood pressure centile charts and implementation of the algorithm for managing increased blood pressure as proposed by the European ADHD Guidelines Group [56]. However, notwithstanding these changes, the core of the DACCP has remained essentially intact since its inception, demonstrating the generalizability of the pathway and the capacity for translation into other healthcare systems.

The DACCP is protocol-driven but flexible. Importantly, the protocols are not profession-specific, allowing best use of the staff available. Nurse-led clinics are clinically- and cost-effective within our setting. In healthcare systems where only doctors are able to manage ADHD, these protocols facilitate rapid training and establish consistent standards of care.

Some elements of the DACCP may not translate into other healthcare systems so easily. For example, the DACCP is strongly multidisciplinary and this brings many benefits. For services where such multidisciplinary working within a clinical team is more difficult, we would suggest discussing opportunities for virtual teams with agreed cross-referral protocols.

Another commonly discussed problem concerns the assessment of psychiatric comorbidities within a non-psychiatric setting. Clinical guidelines are in agreement that integration of assessment of comorbidities into ADHD work-up is essential. To facilitate this, we have successfully trained paediatricians and paediatric nurses to conduct a full mental health assessment, typically using structured and semi-structured interviews such as the Development and Well Being Assessment and K-SADS-PL. Once comfortable and confident with this structured approach they will switch to our systematic (but less structured) assessment protocol described above (Additional file 2). An alternative approach would be to use a screening questionnaire such as the Strengths and Difficulties Questionnaire [57] or Child Behaviour Checklist [58] to identify patients with possible comorbidities and make any necessary arrangements for patients to be further assessed by an appropriately trained specialist.

A further issue concerns the prescription of medications. Unlike in the UK, this may not be delegated to nurses in some countries (although the use of experienced doctors as described above may assist here). Many tasks are already performed by case managers other than the physician, and private practices are encouraged to establish multidisciplinary teams. At the same time, enormous differences in terms of acceptance and treatment approaches continue to exist, not only between European countries, but also between regions within those countries. The sharing of best practice and the creation of treatment pathways based on clinical and scientific evidence could help institutions to improve their standards.

Our clinic documentation and the SKAMP teachers rating scale are available as online Additional files. Alternative documentation is available from the Canadian ADHD Resource Alliance [59]. Their assessment toolkit has many similarities to our own and may be preferred by some clinicians [60].

Administrative aspects to consider when implementing a pathway based on the DACCP principles are the need for a good organization to ensure the necessary forms and instruments are available for distribution, and that systems are in place to follow-up with schools regarding the return of questionnaires and reports.

## Conclusions

The DACCP uses staff skills and time effectively via a structured core pathway to provide a consistent, up-to-date, evidence-based approach to the treatment and management of children and adolescents with ADHD. The DACCP uses standard protocols for the assessment, titration and routine monitoring of clinical care and treatment outcomes. The pathway provides effective care in a real-world setting and has demonstrated success in the long-term management of ADHD. As with any clinical pathway, there are limitations; it is time-intensive and requires well-trained staff. However, we believe that the need for this standard of care is evident and that patients with ADHD should be managed within a pathway that strives for optimal care. While the pathway is continually developing, it has remained essentially intact, demonstrating its flexibility and capacity for translation into other healthcare systems. However, we continually strive to improve the efficiency of our service without compromising clinical standards.

## Additional files

10.1186/s13034-015-0083-2 SKAMP (Swanson, Kotkin, Agler, M-Flynn and Pelham) rating scale form for completion by teachers.
10.1186/s13034-015-0083-2 Clinic assessment document
10.1186/s13034-015-0083-2 Instruments and scales commonly used by staff within the Dundee ADHD Clinical Care Pathway.
10.1186/s13034-015-0083-2 Sample development assessment report.
10.1186/s13034-015-0083-2 ADHD care package clinic documentation.

## Abbreviations

ADHD: attention-deficit/hyperactivity disorder
ADHD-RS-IV: attention-deficit/hyperactivity disorder rating scale IV
CAMHS: Child and Adolescent Mental Health Service
CAPA: Choice and Partnership Approach
DACCP: Dundee ADHD Clinical Care Pathway
HKD: hyperkinetic disorder
ICD: International Classification of Diseases
K-SADS-PL: Schedule for Affective Disorders and Schizophrenia for School-Age Children-Present and Lifetime version
MTA: Multimodal Treatment Study of Children with ADHD
NICE: National Institute for Clinical Excellence
NFPP: New Forest Parenting Programme
NHS: National Health Service
SD: standard deviation
SIGN: Scottish Intercollegiate Guidelines Network
SKAMP: Swanson, Kotkin, Agler, M-Flynn and Pelham scale
SNAP: Swanson, Nolan and Pelham questionnaire

## Footnotes

1 A mental health practitioner who focuses on the interface between primary and secondary care. Primary mental health workers may have a variety of professional backgrounds, including nursing, psychology, social work and education.
