# Supplementary material for: Effective management of attention-deficit/hyperactivity disorder (ADHD) through structured re-assessment: the Dundee ADHD Clinical Care Pathway
Source: Child Adolesc Psychiatry Ment Health. 2015 Nov 19;9:52. doi: 10.1186/s13034-015-0083-2 (PMC4652349; doi:10.1186/s13034-015-0083-2)
Supplement: Supplementary file 1 — 10.1186/s13034-015-0083-2 SKAMP (Swanson, Kotkin, Agler, M-Flynn and Pelham) rating scale form for completion by teachers. [file 13034_2015_83_MOESM1_ESM.docx]

| **Pupil’s name** | Pupil’s date of birth | Pupil’s school |
| --- | --- | --- |
| Please write **your name** here | Please say your relationship to  the pupil:  Class teacher  Head Teacher  Guidance teacher  Support teacher  Other (please say who) | Class |
|  |  | Today’s date |

Please rate the pupil named above thinking about her or his performance in school over the last ___^[[1]](#footnote-1)^*4 weeks. Just circle the number that best represents the pupil’s performance.

| **SKAMP** | | **Not at all** | **Just a little** | **Pretty much** | **Very much** |
| --- | --- | --- | --- | --- | --- |
| 1 | Difficulty getting started on classroom assignments | 0 | 1 | 2 | 3 |
| 2 | Difficulty staying on task for a classroom period | 0 | 1 | 2 | 3 |
| 3 | Problems with interactions with peers in the classroom | 0 | 1 | 2 | 3 |
| 4 | Problems in interactions with staff (teacher or assistant) | 0 | 1 | 2 | 3 |
| 5 | Difficulty remaining quiet according to classroom rules | 0 | 1 | 2 | 3 |
| 6 | Difficulty staying seated according to classroom rules | 0 | 1 | 2 | 3 |
| 7 | Problems in completion of work or classroom assignments | 0 | 1 | 2 | 3 |
| 8 | Problems in accuracy or neatness of written work in  the classroom | 0 | 1 | 2 | 3 |
| 9 | Difficulty attending to an activity or discussion of the class | 0 | 1 | 2 | 3 |
| 10 | Difficulty stopping and making transition to the next period | 0 | 1 | 2 | 3 |

| Please use this space to tell us anything else about the pupil you think we ought to know:  *Continue on a separate sheet if necessary* |
| --- |

#### Thank you for your help

1. * Clinician to write in number of weeks if not 4 [↑](#footnote-ref-1)
