# Supplementary material for: Effective management of attention-deficit/hyperactivity disorder (ADHD) through structured re-assessment: the Dundee ADHD Clinical Care Pathway
Source: Child Adolesc Psychiatry Ment Health. 2015 Nov 19;9:52. doi: 10.1186/s13034-015-0083-2 (PMC4652349; doi:10.1186/s13034-015-0083-2)
Supplement: Supplementary file 2 — 10.1186/s13034-015-0083-2 Clinic assessment document [file 13034_2015_83_MOESM2_ESM.doc]

**Use patient label or**

**DOB _______________________________________**

**Name ________________________________________**

**Today’s date ___________________**

**PRE-SESSION**

**Reason for referral** *(from referral letter)*

**IN-SESSION**

**Appointment attended by**

**Presenting problems** *(from perspective of patient and parents/carers)*

DOB, date of birth

**Global functioning** *(learning disability/development, self-care, school, family, interpersonal/peers, community, forensic)*

**Health and developmental history** *(medical history, psychiatric history, treatment history – include non-NHS, medication, OTs, S&LTs)*

NHS, National Health Service; OT, occupational therapy; S&LT, speech and language therapy

| **ADHD**  A structured assessment of ADHD is conducted using the ADHD sections of the Schedule for Affective Disorders and Schizophrenia for School-Age Children-Present and Lifetime Version (K-SADS-PL; Kaufman J, Birmaher B, Brent D, Rao U and Ryan N. Version 1.0 of October 1996): this comprises questions 1–3 of the ADHD section (pages 36 and 37), and questions 1–16 of the behavioural disorders supplement for ADHD.  This is not presented here for reasons of copyright but is available at: <http://www.psychiatry.pitt.edu/sites/default/files/Documents/assessments/ksads-pl.pdf> | | | | | |
| --- | --- | --- | --- | --- | --- |
|
|
|
| **ADHD Symptom count** | | |  | **Current episode** | **Most severe past** |
|  | **Current episode** | **Most severe past** | Symptom duration > 6 months | 0 1 2 | 0 1 2 |
| Inattention | ………….. | …………… | Age of onset under 7  (write in when: _____________ years ) | 0 1 2 | 0 1 2 |
| Hyperactivity | ………….. | …………… | Impairment – with peers | 0 1 2 | 0 1 2 |
| Impulsivity | ………….. | …………… | Impairment – with family | 0 1 2 | 0 1 2 |
|  | | | Impairment – at school | 0 1 2 | 0 1 2 |

| **Other mental health** | |
| --- | --- |
| **Mood** |  |
| **Anxiety**  *(including suspected or likely OCD symptoms)* |  |
| **Conduct problems** |  |

ADHD, attention-deficit/hyperactivity disorder; OCD, obsessive-compulsive disorder

| **Substance misuse** *(including tobacco, alcohol and solvents)* |  |
| --- | --- |
| **Other mental health**  *(e.g. tics, psychosis, mania)* |  |

**Systemic issues** *(family system, school, social care, juvenile justice, Children’s Reporter, voluntary sector)*

**Other information**

**IMMEDIATELY POST-SESSION**

**Standardised rating scale scores**

| Parent/carer-completed | Teacher-completed |
| --- | --- |
| Conner’s rating Scale (t-scores)   - Oppositional ………………………….... - Cognitive problems/inattention………... - Hyperactivity…………………………… - Conner’s’ ADHD index………………… | Conner’s rating Scale (t-scores)   - Oppositional…………………………..... - Cognitive problems/inattention………… - Hyperactivity…………………………… - Conner’s’ ADHD index………………… |
| Strengths & Difficulties Questionnaire (subscale total)   - Conduct problems………………......... - Hyperactivity……………………......... - Emotional symptoms……………….... - Peer problems……………………....... - Prosocial behaviour……………......... - Total impact score …………….......... | Strengths & Difficulties Questionnaire (subscale total)   - Conduct problems………………......... - Hyperactivity ……………………........ - Emotional symptoms…………………. - Peer problems……………………....... - Prosocial behaviour……………......... - Total impact score ………………...... |
| Social & Communication Questionnaire  Score ………………………. | **CGI–Severity …………..**  **CGAS …………………....** |
| DCD Questionnaire (tick one)   - Indication of DCD….... - Suspect DCD….......... - Probably not DCD….. |

**Summarise school narrative report**

ADHD, attention-deficit/hyperactivity disorder; CGAS, Clinical Global Assessment Scale; CGI, Clinical Global Impressions; DCD, Developmental Coordination Disorder

**Clinical impression**

| **Unanswered questions**     | **ASSESSOR SIGNATURE DATE**    **NAME IN CAPS** | | --- |   **Action plan**   - - Obtain school report and questionnaires   - Obtain previous school reports   - BPVS   - Educational Psychology report   - CHATTI   - School observation   - Review Community Child Health notes   - SW liaison   - SLT report/referral   - OT report/referral   - Other (please specify) |
| --- | --- |

BPVS, British Picture Vocabulary Scale; CHATTI, Child Attention-Deficit/Hyperactivity Disorder Teacher Telephone Interview; OT, occupational therapy; SLT, speech and language therapy; SW, social worker
