# Supplementary material for: Effective management of attention-deficit/hyperactivity disorder (ADHD) through structured re-assessment: the Dundee ADHD Clinical Care Pathway
Source: Child Adolesc Psychiatry Ment Health. 2015 Nov 19;9:52. doi: 10.1186/s13034-015-0083-2 (PMC4652349; doi:10.1186/s13034-015-0083-2)
Supplement: Supplementary file 3 — 10.1186/s13034-015-0083-2 Instruments and scales commonly used by staff within the Dundee ADHD Clinical Care Pathway. [file 13034_2015_83_MOESM3_ESM.docx]

Instruments and scales commonly used by staff within the Dundee ADHD Clinical Care Pathway

| **Instrument** | **Reference** | **Purpose** |
| --- | --- | --- |
| K-SADS-PL | Kaufman et al., 1996 [1] | Semi-structured diagnostic interview for a wide range of  psychopathologies, including; ADHD, psychoactive disorders, depression, obsessive compulsive disorder |
| Developmental and Wellbeing Assessment (DAWBA) | Goodman et al., 2000 [2] | Modular, structured assessment interview of comorbid psychiatric disorders |
| Social Communication Questionnaire (SCQ) | Rutter et al., 2003 [3] | Screening questionnaire for autism spectrum disorders |
| Developmental Coordination Disorders Questionnaire 2007 (DCDQ 2007) | Wilson et al., 2009 [4] | Screening questionnaire for developmental coordination disorders |
| British Picture Vocabulary Scale | Dunn et al. [5] | Screen for verbal performance (proxy measure for verbal IQ) |
| Short Mood and Feelings Questionnaire (SMFQ) | Angold et al., 1995 [6] | Screen and outcome measure for depression |
| Screen for Child Anxiety Related Disorders (SCARED) | Birmaher et al., 1999 [7] | Screen and outcome measure for anxiety disorders |
| Yale Global Tic Severity Scale (YGTSS) | Leckman et al., 1989 [8] | Outcome measure for tic disorders |
| Child’s Sleep Habits Questionnaire (CSHQ) | Owens et al., 2000 [9] | Assessment and outcome measure for sleep problems |
| Weiss Functional Impairment Rating Scale (WFIRS) | Available from <http://www.caddra.ca/pdfs/caddraGuidelines2011_Toolkit.pdf>* | Outcome measure for ADHD related impairments |
| Child Health Illness Profile (CHIP) | Riley et al., 2004 [10] | Outcome measure for quality of life |
| Dundee Difficult Times of Day (D-DTODS) | Available from <http://www.healthcareimprovementscotland.org/our_work/mental_health/adhd_services_over_scotland/stage_3_adhd_final_report.aspx> | Tool for assessing ADHD symptoms across the day |
| Dundee Blood Pressure Charts | Available from <http://www.healthcareimprovementscotland.org/our_work/mental_health/adhd_services_over_scotland/stage_3_adhd_final_report.aspx> | Blood Pressure scales based on UK norms with 95th centile |

*The CADDRA website also contains a range of other helpful instruments and scales

**References**

1. Kaufman J, Birmaher B, Brent D, Rao U, Ryan N. Diagnostic Interview. Kiddie-Sads-Present and Lifetime Version (K-SADS-PL). Version 1.0 of October 1996. [<http://www.psychiatry.pitt.edu/sites/default/files/Documents/assessments/ksads-pl.pdf>]

2. Goodman R, Ford T, Richards H, Gatward R, Meltzer H. The Development and Well-Being Assessment: description and initial validation of an integrated assessment of child and adolescent psychopathology. J Child Psychol Psychiatry. 2000;41:645-55.

3. Rutter M, Bailey A, Lord C. Social Communication Questionnaire (SCQ). Torrance, CA: Western Psychological Services; 2003.

4. Wilson BN, Crawford SG, Green D, Roberts G, Aylott A, Kaplan BJ. Psychometric properties of the revised Developmental Coordination Disorder Questionnaire. Phys Occup Ther Pediatr. 2009;29:182-202.

5. Dunn L, Dunn L, Whetton C, Burley J. British Picture Vocabulary Scale. London: NFER-Nelson; 1997.

6. Angold A, Costello EJ, Messer S, Pickles A. Development of a short questionnaire for use in epidemiological studies of depression in children and adolescents. Int J Methods Psychiatr Res. 1995;54:237-49.

7. Birmaher B, Brent DA, Chiappetta L, Bridge J, Monga S, Baugher M. Psychometric properties of the Screen for Child Anxiety Related Emotional Disorders (SCARED): a replication study. J Am Acad Child Adolesc Psychiatry. 1999;38:1230-6.

8. Leckman JF, Riddle MA, Hardin MT, Ort SI, Swartz KL, Stevenson J, et al. The Yale Global Tic Severity Scale: initial testing of a clinician-rated scale of tic severity. J Am Acad Child Adolesc Psychiatry. 1989;28:566-73.

9. Owens JA, Spirito A, McGuinn M. The Children's Sleep Habits Questionnaire (CSHQ): psychometric properties of a survey instrument for school-aged children. Sleep. 2000;23:1043-51.

10. Riley AW, Forman EM, Starfield B, Rebok GW, Robertson JA, Green BF. The Parent Report Form of the CHIP-Child Edition: reliability and validity. Med Care. 2004;42:210-20.
