# Supplementary material for: Effective management of attention-deficit/hyperactivity disorder (ADHD) through structured re-assessment: the Dundee ADHD Clinical Care Pathway
Source: Child Adolesc Psychiatry Ment Health. 2015 Nov 19;9:52. doi: 10.1186/s13034-015-0083-2 (PMC4652349; doi:10.1186/s13034-015-0083-2)
Supplement: Supplementary file 4 — 10.1186/s13034-015-0083-2 Sample development assessment report. [file 13034_2015_83_MOESM4_ESM.doc]

**DEVELOPMENTAL ASSESSMENT REPORT**

**DATE** _________________

Patient’s name __________________________________

Patient’s address________________________________________________________________________________________________________________________________

**Reason for referral**

*Patient A,* an 8 year old boy, was referred by his general practitioner (GP) due to concerns regarding his overactivity, impulsivity and emotion regulation. His parents had been asked by the school to visit the GP as they felt that *Patient A* was struggling in school. Despite support, *Patient A* was spending increasing amounts of time out of class as he was disruptive. He would have difficulty committing to tasks for more than a few moments, and could not work independently as he would be off task or disrupting others. He would wander around the classroom and had been found wandering around the school on several occasions. He required additional supervision in the playground as he could be too boisterous and could place himself at risk due to impulsivity; he had recently run out of the school grounds, and his school were considering asking his parents to take him home for lunch to try and reduce the risk of this happening again. It was also noted that *Patient A*’s attention was very variable, coping better when working in pairs or in small groups. He found sitting still very difficult and was constantly fiddling and fidgeting. His parents were concerned that *Patient A’s* behaviour was due to their recent separation and were keen to get a more comprehensive assessment of *Patient A’s* wellbeing, particularly as *Patient A* would be going into Year 4 of primary school after the summer holidays.

**Nature of assessment**

Initially, *Patient A* was seen at a screening appointment in Jan 2013 to try and clarify the reasons for his difficulty, and to determine which of the teams within the service would best meet his needs.

Following this appointment, *Patient A* was placed on the waiting list for a comprehensive developmental and mental health assessment.

I met *Patient A* and both his parents on two occasions to complete the assessment. Despite the separation, both parents attended both appointments. They provided *Patient A’s* school reports for Years 1–3. *Patient A’s* primary school also returned completed SKAMP[[1]](#footnote-2) and narrative forms.

**Presenting problems**

Mum and Dad reported that they had always felt *Patient A* had “lots of energy”, but this had not caused any particular difficulties at nursery school. The nursery had consistently reported that *Patient A* was “like a whirlwind”. They noted that he was popular with peers and tended to like much more physical activities. He found it difficult to sit for any length of time and any drawings he did tended to be brief scribbles. His parents were advised that there was no need to consider delaying his transition to primary school as the increased structure at primary school could be helpful. His Year 1 teacher noted that *Patient A* was a “lovely, bubbly boy with lots of enthusiasm”. He struggled with quiet activities and could find it difficult to listen. His Year 2 teacher found similar difficulties and noted that he was always keen to take part in class discussions but struggled more when it came to writing things down. He remained with the same teacher in Year 3 and, while there were some improvements in his ability to wait, he remained a ‘”handful”’ and he worked best when he sat near her and was given very small amounts of work to do at any one time. He struggled more when there was any change in teacher or in less structured activities, like music or gym.

At the time of his first assessment appointment, the school was using a daily target system, which was being reinforced at home. His current targets of ‘to put my hand up before I speak’, ‘to sit on the floor at story time’ and ‘to walk between classrooms’ were of limited success in helping him manage his behaviour.

**General functioning**

Most days *Patient A* gets up and dresses himself with a little help from Mum (his clothes are laid out for him the night before and Mum made a visual chart to help him remember in what order his clothes go on). Mum reported that most days she has to repeatedly prompt him with this, despite the incentive of being allowed to play with his toys or watch TV when he is dressed. When eating breakfast he appears to be in a “daydream”. He walks to school with Mum, which can be very stressful, as *Patient A* will tend to dash ahead and there are several busy roads to cross. Mum feels that he has very little awareness of road safety and his parents must still hold his hand when they are crossing the road or in car parks.

After school, he occasionally goes out with friends, but Mum said she usually preferred to have him play at home with friends so she could supervise him better as he could occasionally fall out with friends if they would not play the games he wanted.

**Brief family and social history**

*Patient A* lives with his mother, Mrs W, a shop manager, his older sister, B, aged 14 years, and younger sister, M, aged 5 years. Mum and Dad reported that they had separated 8 months ago and that this was an amicable separation. All the children continued to spend time with Dad, staying over at his house on alternate weekends. Mr W worked as a sales manager and was often away during the week. This had been the case for some time, so the parents felt that the children were less unsettled by their separation than they might have been otherwise.

*Patient A* had considerable contact with his maternal grandparents, who often helped out with childcare after school and in the school holidays. *Patient A* also had regular contact with his paternal grandparents.

**Birth**

Mrs W reported no problems during her pregnancy or the birth. She is a non-smoker and denied any medication, drug or alcohol use during pregnancy. *Patient A* was born at 39 weeks gestation by SVD[[2]](#footnote-3), weighed 7lb 12oz and did not require any time in the SCBU[[3]](#footnote-4). Mum described experiencing some mild postnatal symptoms of depression but did not require antidepressants.

**Development milestones and physical health**

There were no reported concerns regarding *Patient A’s* milestones. He started to walk at around 13 months of age, and speech was normal. His first words were spoken when he was around 12–15 months of age.

**Enuresis/encopresis/constipation**

He was fully toilet trained during the day by 3 years of age and was dry through the night by 4 years of age.

**Appetite**

There were no concerns about *Patient A’s* growth and he is reported to have a good appetite and like a varied diet, eating a wide range of foods. When asked if he got up from the table during meals, he was quite shocked and responded that he never left the table “if I have lunch in front of me”.

**Vision**

No problems.

**Hearing**

No problems (passed routine hearing screen).

**Sleep**

*Patient A* goes to bed between 8 and 8.30 pm during the week. It usually takes him about an hour to settle, but he has no problems getting up for school.

**Fine/gross motor skills**

*Patient A* is reported to be quite clumsy when walking and trips up when running. Mrs W usually carries *Patient A’s* food to the table for him as he often drops things. She reports that she usually cuts up his food as “he can’t do that and he makes a mess”. Talking this through, it seems that these difficulties may be due to *Patient A* rushing tasks and not looking where he is going. We have agreed, however, that we would keep this under review and refer to occupational therapy if there were ongoing concerns about fine or gross motor skills.

**Temperament**

Mum and Dad thought that, apart from the over-activity and poor concentration, *Patient A* was quite an easygoing child but felt that he liked to have his routines and they were finding that he was a little more defiant lately.

**Clinical findings**

**Mental health assessment**

**Appearance and behaviour**

*Patient A* presented as a friendly young man, with a sense of humour, who clearly found it difficult to remain focused within the clinic. His eye contact was good, although he was easily distracted and tended to flit between toys in the clinic. His speech was clear, but it was reported that *Patient A* could find it hard to relay a story in a sequential way, often jumping from one point to another.

**Cognitive**

*Patient A’s* standardized score on the BPVS[[4]](#footnote-5) was 82, giving him a percentile rank of 12 and an age equivalent of 8 years.

**ADHD**

His ADHD[[5]](#footnote-6) symptoms were assessed using the K-SADS-PL,[[6]](#footnote-7) a semi structured diagnostic interview.

ADHD symptom report:

Inattention: 8

Hyperactivity: 5

Impulsivity: 3

Age of onset: noticeable since a toddler but not impairing until he started primary school.

Impairment is present with peers, family and school. Other parents are starting to be reluctant to let their children play with *Patient A* as he is perceived as being “a bit wild”. *Patient A* is noticing that he is not being invited to parties and expresses sadness about this. *Patient A* is reported to make friends easily but can be easily led. Other family members are commenting on *Patient A’s* behaviour and the school are suggesting that his parents take him home at lunchtime, which would cause considerable difficulties for Mum with work.

**SKAMP completed by Year 3 teacher**

Inattention scale mean score: 1.8

Deportment scale mean score: 2.3

**Social communication questionnaire**

A total score of 6 does not suggest that further screening for pervasive developmental disorder is indicated.

**Mood**

*Patient A* reports that he can sometimes feel sad, although this doesn’t usually last much longer than an hour or so. His parents report that *Patient A* is generally a happy little boy.

**Anger/ODD[[7]](#footnote-8)**

*Patient A* has had very minor issues with temper outbursts for a number of years. Mrs W reported that he has been a bit cheekier lately, but they are managing this with minor sanctions for defiance or talking back, which seems to be helping. He is not spiteful or vindictive, and there is no history of any more significant disruptive behaviour.

**Anxiety/OCD[[8]](#footnote-9)**

*Patient A* reported that he likes having two bedrooms now (one at Mum’s and one at Dad’s). He sometimes worries about Dad as he knows that he often has to drive at night.

*Patient A* reports having some generalized worries. He has some worries that his friends don’t like him and they sometimes talk about playing together after school when he is not invited.

He reported that he is good at football and his best friend is *Child A*.

Mum reports that *Patient A* wants to have friends and likes to be part of a group. He spends time playing football with a group of boys.

**Trauma**

There is no history of *Patient A* experiencing any traumatic events. His parents’ separation does not appear to have caused any significant concern for *Patient A.*

**Summary of external reports**

**Information from [insert name] primary school**

Reports from Years 1–3 were reviewed.

These consistently mention that *Patient A* is a very active little boy who likes to chat. Within these reports there were references to *Patient A* needing support to stay focused, plan his work, finish his work on time and needing prompting to carry out tasks. He was repeatedly reported to have problems with concentration, being easily distracted and distracting others. There is also reference to *Patient A* not completing homework and being behind for his age.

It is noted by his Year 3 teacher that she thinks *Patient A* is not performing to his ability level; his reluctance to put pen to paper means that his current work output is likely to be an underestimate of his true abilities.

**Goals and expectations**

*Patient A*’s parents wanted to make sure that they were doing their best to help *Patient A*. They had worried that their separation was causing difficulties for *Patient A* but by going through the assessment process they could see that *Patient A’s* difficulties pre-dated their separation. In addition, they could see how well they were actually managing *Patient A’s* behaviour and that they had naturally adapted the strategies that they used to take into account *Patient A’s* ADHD symptoms. They were concerned about what the future might hold for *Patient A* and wanted *Patient A* to achieve to the best of his potential. They were willing to consider any supports or interventions, including medication, which might be helpful for *Patient A.*

**Provisional m**ulti-axial diagnosis

| Axis I – mental illness | ADHD |
| --- | --- |
| Axis II – specific developmental disorders |  |
| Axis III – learning disability |  |
| Axis IV – physical health |  |
| Axis V – abnormal psychosocial factors |  |
| Axis VI – global functioning |  |

CGAS[[9]](#footnote-10) – 55 CGI[[10]](#footnote-11) – 4

**SIGNATURE DATE:** 19 December 2013

**NAME**

**JOB TITLE**

1. SKAMP, Swanson, Kotkin, Agler, M-Flynn and Pelham scale. [↑](#footnote-ref-2)
2. SVD, Spontaneous Vaginal Delivery. [↑](#footnote-ref-3)
3. SCBU, Special Care Baby Unit. [↑](#footnote-ref-4)
4. BPVS, British Picture Vocabulary Scale. [↑](#footnote-ref-5)
5. ADHD, attention-deficit/hyperactivity disorder. [↑](#footnote-ref-6)
6. K-SADS-PL, Schedule for Affective Disorders and Schizophrenia for School-Age Children-Present and Lifetime Version. [↑](#footnote-ref-7)
7. ODD, oppositional defiant disorder. [↑](#footnote-ref-8)
8. OCD, obsessive-compulsive disorder. [↑](#footnote-ref-9)
9. CGAS, Clinical Global Assessment Scale. [↑](#footnote-ref-10)
10. CGI, Clinical Global Impressions. [↑](#footnote-ref-11)
