# Supplementary material for: Effective management of attention-deficit/hyperactivity disorder (ADHD) through structured re-assessment: the Dundee ADHD Clinical Care Pathway
Source: Child Adolesc Psychiatry Ment Health. 2015 Nov 19;9:52. doi: 10.1186/s13034-015-0083-2 (PMC4652349; doi:10.1186/s13034-015-0083-2)
Supplement: Supplementary file 5 — 10.1186/s13034-015-0083-2 ADHD care package clinic documentation. [file 13034_2015_83_MOESM5_ESM.docx]

| **Appointment type (circle one)** | | | |
| --- | --- | --- | --- |
| Hello | Titration | Continuing Care | Asked to see |

**Clinicians**: Doctor…………………………………………… Nurse ……….…………………………………………….

**Others:** …………………………………………………………………...

**Appointment Attended by**: …………………………………………………………….  **DNA** □

| Medication *(if none, write none)* | | | | |
| --- | --- | --- | --- | --- |
| **Medication name** | **Dose 1** | **Dose 2** | **Dose 3** | **Dose 4** |
|  |  |  |  |  |
|  |  |  |  |  |

| Overall progress and problems | |
| --- | --- |
| Parent/carer view |  |
| Child view |  |
| Information from school (end of term report, feedback, meetings, etc) |  |

| Variables | | | | | |
| --- | --- | --- | --- | --- | --- |
| 10-item Conners 3  t-scores | |  | | Physiological variables | |
| Parent version |  |  |  | Height | cm |
| Teacher version |  |  |  | Weight | kg |
|  |  |  |  | Blood pressure | mmHg |
|  |  |  |  | Pulse |  |

DNA, did not attend; DOB, date of birth **SWANSON, NOLAN & PELHAM (SNAP IV)**

Clinician-scored oppositional defiant disorder (ODD), Inattention, Hyperactivity/Impulsivity

(Reproduced by kind permission of Dr J Swanson. All rights reserved.

Any reuse requires permission from Dr J Swanson)

| **OPPOSITIONALITY (ODD)** | | Never or rarely (never) | Sometimes (mild) | Often (moderate) | Very often (severe) |
| --- | --- | --- | --- | --- | --- |
| 1 | Often loses temper | 0 | 1 | 2 | 3 |
| 2 | Often argues with adults | 0 | 1 | 2 | 3 |
| 3 | Often actively defies or refuses adult requests or rules | 0 | 1 | 2 | 3 |
| 4 | Often deliberately does things that annoy other people | 0 | 1 | 2 | 3 |
| 5 | Often blames others for his or her mistakes or misbehaviour | 0 | 1 | 2 | 3 |
| 6 | Often touchy or easily annoyed by others | 0 | 1 | 2 | 3 |
| 7 | Often is angry and resentful | 0 | 1 | 2 | 3 |
| 8 | Often is spiteful or vindictive | 0 | 1 | 2 | 3 |

**ODD TOTAL SCORE: = ODD SUMMARY SCORE (TOTAL SCORE / 8) =**

| **INATTENTION (INATT)** | | Never or rarely (never) | Sometimes (mild) | Often (moderate) | Very Often (severe) |
| --- | --- | --- | --- | --- | --- |
| 9 | Fails to give close attention to details or makes careless mistakes in schoolwork | 0 | 1 | 2 | 3 |
| 10 | Has difficulty sustaining attention in tasks or play activities | 0 | 1 | 2 | 3 |
| 11 | Does not seem to listen when spoken to directly | 0 | 1 | 2 | 3 |
| 12 | Does not follow through on instructions and fails to finish schoolwork, chores or duties | 0 | 1 | 2 | 3 |
| 13 | Has difficulty organising tasks and activities | 0 | 1 | 2 | 3 |
| 14 | Avoids tasks (e.g. schoolwork, homework) that requires sustained mental effort | 0 | 1 | 2 | 3 |
| 15 | Loses things necessary for tasks or activities (e.g. toys, school assignments, pencils or books) | 0 | 1 | 2 | 3 |
| 16 | Is easily distracted | 0 | 1 | 2 | 3 |
| 17 | Is forgetful in daily activities | 0 | 1 | 2 | 3 |

**INATT TOTAL SCORE: = INATT SUMMARY SCORE (TOTAL SCORE / 9) =**

| **HYPERACTIVITY/IMPULSIVITY (HYP/IMP)** | | Never or rarely (never) | Sometimes (mild) | Often (moderate) | Very Often (severe) |
| --- | --- | --- | --- | --- | --- |
| 18 | Fidgets with hands or feet or squirms in seat | 0 | 1 | 2 | 3 |
| 19 | Leaves seat in classroom or in other situation in which remaining seated is expected | 0 | 1 | 2 | 3 |
| 20 | Runs about or climbs excessively in situations in which it is inappropriate | 0 | 1 | 2 | 3 |
| 21 | Has difficulty playing or engaging in leisure activities quietly | 0 | 1 | 2 | 3 |
| 22 | Is “on the go” or acts as if “driven by a motor” | 0 | 1 | 2 | 3 |
| 23 | Talks excessively | 0 | 1 | 2 | 3 |
| 24 | Blurts out answers before questions have been completed | 0 | 1 | 2 | 3 |
| 25 | Has difficulty waiting turn | 0 | 1 | 2 | 3 |
| 26 | Interrupts or intrudes on others | 0 | 1 | 2 | 3 |

**HYP/IMP TOTAL SCORE: = HYP/IMP SUMMARY SCORE (TOTAL SCORE / 9) =**

| Other symptoms | | | | | |
| --- | --- | --- | --- | --- | --- |
|  | Not present | Present but not impairing | Present and impairing | Present and severely impairing | Write note↓ |
| Insomnia or trouble sleeping | 0 | 1 | 2 | 3 |  |
| Nightmares | 0 | 1 | 2 | 3 |  |
| Drowsiness | 0 | 1 | 2 | 3 |  |
| Nausea | 0 | 1 | 2 | 3 |  |
| Anorexia (Less hungry than other children) | 0 | 1 | 2 | 3 |  |
| Stomach-aches | 0 | 1 | 2 | 3 |  |
| Headaches | 0 | 1 | 2 | 3 |  |
| Dizziness | 0 | 1 | 2 | 3 |  |
| Sad/unhappy | 0 | 1 | 2 | 3 |  |
| Prone to crying | 0 | 1 | 2 | 3 |  |
| Irritable | 0 | 1 | 2 | 3 |  |
| Thoughts of self-harm | 0 | 1 | 2 | 3 |  |
| Suicidal ideation | 0 | 1 | 2 | 3 |  |
| Euphoric/unusually happy | 0 | 1 | 2 | 3 |  |
| Anxious | 0 | 1 | 2 | 3 |  |
| Tics or nervous movements | 0 | 1 | 2 | 3 |  |
| “Spaced-out”/“Zombie-like” | 0 | 1 | 2 | 3 |  |
| Less talkative than other children | 0 | 1 | 2 | 3 |  |
| Less sociable than other children | 0 | 1 | 2 | 3 |  |

| **Medication-related problems** | | | | | |
| --- | --- | --- | --- | --- | --- |
|  | Yes | No | Not assessed | Write note↓ | |
| Morning breakthrough symptoms | □ | □ | □ |  | |
| Afternoon breakthrough symptoms | □ | □ | □ |  | |
| Patient-related compliance problems | □ | □ | □ |  | |
| Parent/carer-related compliance problems | □ | □ | □ |  | |
| School-related compliance problems | □ | □ | □ | Mean doses missed/week  at school ____________ |  |
| Ridicule/stigma | □ | □ | □ |  | |
| Restriction of activities | □ | □ | □ |  | |
| Diversion | □ | □ | □ |  | |
| Other | □ | □ | □ |  | |

| **Current comorbid disorders/clinical problems** | | | | | |
| --- | --- | --- | --- | --- | --- |
|  | Yes | No | Not assessed | New | Write note↓ |
| ODD/CD | □ | □ | □ | □ |  |
| Anxiety (including OCD) | □ | □ | □ | □ |  |
| Depression | □ | □ | □ | □ |  |
| Tics/Tourette’s Syndrome | □ | □ | □ | □ |  |
| Tobacco use | □ | □ | □ | □ |  |
| Alcohol use | □ | □ | □ | □ |  |
| Cannabis use | □ | □ | □ | □ |  |
| Other drug use | □ | □ | □ | □ |  |
| Sleep problems | □ | □ | □ | □ |  |
| Epilepsy/seizures | □ | □ | □ | □ |  |
| Other | □ | □ | □ | □ |  |

CD, conduct disorder; OCD, obsessive-compulsive disorder; ODD, oppositional defiant disorder

| **Clinical global impressions** | | | | | |
| --- | --- | --- | --- | --- | --- |
| CGI severity |  | CGI change |  | CGAS |  |

| **Psychosocial interventions planned or recommended** | | | |
| --- | --- | --- | --- |
|  | Yes | No | **Summary and plan** |
| Counselling/psychoeducation | □ | □ |  |
| Parent management training | □ | □ |  |
| Other psychotherapy (say what) | □ | □ |  |
| Liaise with other agency (say who) | □ | □ |  |
| Refer to other agency or NHS speciality (say who) | □ | □ |  |
| Refer for investigations (say what) | □ | □ |  |

| Medication recommended *(if none, write none)* | | | | |
| --- | --- | --- | --- | --- |
| **Medication name** | **Dose 1** | **Dose 2** | **Dose 3** | **Dose 4** |
|  |  |  |  |  |
|  |  |  |  |  |
| Titrate onto: | |  | |  |

| **Follow up** | |
| --- | --- |
| Review in Continuing Care Clinic in | ……………… months |
| Other review |  |
| Discharge to |  |
| Consent for copy letter to Community Child Health Service | □ |

| **Clinician signature** | **Date** |
| --- | --- |
|  |  |

CGAS, Clinical Global Assessment Scale; CGI, Clinical Global Impressions; NHS, National Health Service
